# COVID-19 risk perception and tourist satisfaction: A mixed-method study of the roles of destination image and self-protection behavior

**DOI:** 10.3389/fpsyg.2022.1001231

**Published:** 2022-09-06

**Authors:** Bin Zhou, Si-yi Liu, Ling-en Wang, Lu-ting Wang, Yu-xin Wang

**Affiliations:** ^1^Marine Economics Research Center, Donghai Academy, Ningbo University, Ningbo, China; ^2^Department of Tourism, Ningbo University, Ningbo, China; ^3^Institute of Geographic Sciences and Natural Resources Research, Chinese Academy of Sciences, Beijing, China

**Keywords:** COVID-19, risk perception, destination image, self-protection behavior, tourist satisfaction, China, mixed-method

## Abstract

This study aimed to examine the effects of COVID-19 risk perception on negative destination image and self-protection behavior, and the resultant effects on tourist satisfaction. Hence, this study applied a continuous interpretive mixed-method design combining quantitative and qualitative analyses. A quantitative survey (*n* = 486) in the cities of Ningbo, Huangshan, and Chengdu, China, and 19 qualitative interviews were conducted online. The results of the quantitative study show that: (1) Risk perception and negative destination image are antecedent variables influencing tourist satisfaction, and (2) there are significant positive correlations between risk perception and negative destination image, risk perception and tourist self-protection behavior, and negative destination image and tourist self-protection behavior. Moreover, (3) negative destination image had a partial mediating effect between risk perception and satisfaction. Furthermore, to supplement the research data and expand the quantitative findings, this study further examined whether the above variables are related to tourist satisfaction, through in-depth interviews with tourists. The findings showed that COVID-19 risk perception, negative destination image, and self-protection behavior all affect tourist satisfaction. The findings provide valuable crisis management suggestions for the government and should contribute to the efforts of tourist destinations to build a healthy and safe image, thereby contributing to the sustainable development of tourism industries in the post-epidemic era.

## Introduction

COVID-19 is a new variant of coronavirus infection that commenced in 2019 (Bhati et al., 2021). In 2020, this infection swept the world causing over 1.92 million deaths. As of this writing, the number of COVID-19 infections worldwide exceeded 513 million and deaths had surpassed 6.24 million (WHO, 2022). Its spread was aided by systems of travel and mobility created in part by the tourism industry, and by the initial lack of vaccines. The World Health Organization declared a global pandemic on March 11, 2020. Unlike the effects of natural disasters such as earthquakes, floods, and fires, COVID-19 may cause long-term effects and harm, and is thought likely to recur even after the pandemic is over (Bae and Chang, 2021).

In addition to infections and deaths, the virus has adversely affected the global economy and employment (Chen et al., 2022). Tourism is no exception. With the rapid spread of COVID-19 globally, constrained by containment measures, health and hygiene regulations, the closure of borders, and the grounding of aircraft, domestic and international tourism stalled. In several countries, accommodation, catering, and other tourism-related industrial activities were suspended, and the entire tourism industry was affected (Connor, 2020; Ahmad et al., 2021; Aiello et al., 2022). In the past 2 years, the provision of vaccines has made the global pandemic more controllable in some countries, and various international tourism organizations and scholars have also made recommendations for a pathway to global recovery, yet new virus variants (e.g., Delta and Omicron) remain highly infectious and have spread worldwide. Hence, COVID-19 continues to affect the long-term recovery of tourism (Yang et al., 2021; Zopiatis et al., 2021; Gössling and Schweiggart, 2022).

The risk of disease is a significant matter of concern to international travelers (Kozak et al., 2007). When tourists make travel decisions under conditions of uncertain risks, they may seek to avoid destinations thought unsafe (Beirman, 2002). Tourist satisfaction is an important concept within tourism marketing and has been fruitfully studied by scholars (Kozak and Rimmington, 2000; Nield et al., 2000; Del Bosque and San Martín, 2008; Song et al., 2012). Among them is the relationship between disease risk perception and tourist satisfaction (Li et al., 2016; Huang et al., 2020; Xie et al., 2020). Li et al. (2016) and Xie et al. (2020) have shown that disease risk perception is an important factor influencing tourist satisfaction. Previous studies examining the effects of risk perception on tourist satisfaction have focused on quantitative methods such as regression models and structural equation modeling (Promsivapallop and Kannaovakun, 2017; Alcántara-Pilar et al., 2018; Saayman et al., 2018), and research data are often obtained using cross-sectional methods. At the same time, there are no longitudinal studies associated with visitor interviews to validate the results of quantitative analyses. Furthermore, research on the relationship between risk perception and tourist satisfaction in the context of COVID-19, which has lasted for almost 3 years, is yet to be conducted in depth. This research gap inspired this study, which sought to investigate the evolution of tourists’ risk perceptions, destination image, and self-protection behaviors during post-outbreak travel based on a protection motivation theory (PMT) framework, and the extent to which these factors influenced tourist satisfaction.

This study used a sequential explanatory mixed-methods approach. The questionnaire method and structural equation modeling (SEM) were first used to quantify the impact of risk perception, negative destination image, and self-protection behavior on tourist satisfaction, and additional interview data were collected through in-depth interviews with tourists to allow us to focus on the constant changes that tourist satisfaction presents over time. Thus, this research is a useful addition to studies that statistically focus on the effects of risk perceptions on tourist satisfaction (Yüksel and Yüksel, 2007; Sohn et al., 2016). Another aspect of this research is that it can also help clarify that combining tourist risk perception, negative destination image, and self-protection behavior on changes in tourist satisfaction over time and as COVID-19 continues, is important for the marketing departments of tourist destinations.

## Literature review and hypothesis development

### Protection motivation theory

Protection motivation theory (PMT) was developed by Rogers to explain the phenomenon of individuals adopting protective behaviors in the face of health-related risks (Rogers, 1975). Modified PMT is more general in that preventive behavioral decisions are due to the protective motivation of individuals in response to threats (Rogers, 1983). The theory has been developed in many studies, not only in the field of public health (Conner and Norman, 2015), but also in healthy lifestyle adoption (Scarpa and Thiene, 2011), disease prevention (Eppright et al., 1994), and so on. Meanwhile, some scholars (Horng et al., 2014; Wang et al., 2019; Ruan et al., 2020) applied PMT to the discipline of tourism. Additionally, some researchers used the PMT framework to explain the protective motivation and behavior of individuals in the context of COVID-19, for example, vaccination intention (Wang et al., 2022), dining behavior (Wen and Liu-Lastres, 2022), international travel protection motivation (Qiao et al., 2022), and hotel employee protection motivation (Ghaderi et al., 2022). Therefore, based on a review of previous literature, this study argues that PMT is a good and evolving framework and that the relationship between the constructs (e.g., threat appraisal, coping appraisal, and behavioral intentions) has been tested by several scholars in different contexts. Among them, risk perception (containing two dimensions of perceived severity and perceived vulnerability) has been one of the focal points of scholars’ attention for the behavioral changes that can explain the behavioral changes of tourists from the outbreak phase to the new normal epidemic prevention and control phase. This is especially the case in the face of the major challenges of the current pandemic, in which tourism research and industry practices consider health-protective behavior as a prerequisite for safe travel (Bhati et al., 2021).

### Tourists’ risk perception

The concept of perceived risk originated in the field of psychology and was first introduced by Bauer (1967) into consumer behavior research as a crucial determinant of consumer attitudes and behavior. During a period of a global pandemic, risk perception is considered a robust theory to explain tourists’ behavior and has thus garnered much attention (Shin and Kang, 2020; Bae and Chang, 2021; Foroudi et al., 2021; Godovykh et al., 2021; Kim and Kang, 2021; Sánchez-Cañizares et al., 2021). Perceived risk is subjective and is influenced by an individual’s judgment of the probability of a risky event occurring and his/her social and cultural background (Becken et al., 2017).

Tourism researchers have extensively examined the influencing factors, consequences, and formation mechanism of risk in travel (Cui et al., 2016). The factors influencing perceived risk can be classified under three headings. The first includes the impact of the mode of infection transmission, the severity of the outbreak (number of infections and deaths), the duration of the outbreak, media coverage, government measures, and public opinion (Smith, 2006; Chen et al., 2021). For example, Smith (2006) reported that during the SARS pandemic, people perceived levels of risk higher than was necessary, and the primary reason for this was a limited public understanding of SARS identification and control measures, significant uncertainty regarding the potential negative outcomes, and an over-assessment of interpersonal transmission. Taken together, in some locations, sections of the populace came close to panic (Smith, 2006). Such a sense reflects the second category. This comprises emotional factors, related to personality traits, including tolerance of risk, optimism–pessimism, and perceived control (Cui et al., 2016; Zambrano-Cruz et al., 2018). The third category comprises individual visitor characteristics, such as culture, education, age, and gender (Kuang et al., 2020; Zhan et al., 2022). In addition, some studies have described the impact of perceived risk on tourists’ attitudes and behaviors. For example, it is thought that perceived risk can lead to tourists having negative perceptions about a destination (Alvarez and Campo, 2014). These closely correlate with destination image, self-protection behavior, willingness to pay, satisfaction, and loyalty (Lin et al., 2012; Tavitiyaman and Qu, 2013; Casidy and Wymer, 2016; Caber et al., 2020). Finally, at the theoretical level, other studies have used psychological distance, explanatory level theory, ethnocentrism, and the theory of planned behavior to develop mechanisms that explain tourists’ risk perception during the COVID-19 pandemic of 2020 (Kock et al., 2020; Li et al., 2020; Bae and Chang, 2021).

### Negative destination image

The image of a destination is a psychological representation of an individual’s impressions and beliefs about a particular place (Crompton, 1979). Previous studies have focused on the antecedents that shape destination image, and have identified that a tourist’s characteristics (e.g., psychology and culture) and stimulating factors (including information source and experience) will determine perceptions (Baloglu, 2000; Beerli and Martin, 2004). In addition, the short-term image of a destination is easily affected by public crises such as natural disasters and infectious diseases. If, however, tourists believe that the area is recovering quickly from a disaster, a positive image may be reinforced. Conversely, slow recovery or tourist uncertainty about the status of recovery might create a negative impression. Several studies testify to changes in destination image after an outbreak like SARS, avian flu, AIDS, Ebola, and other pandemic crises (Carter, 1998; Kozak et al., 2007; Rittichainuwat and Chakraborty, 2009; Novelli et al., 2018).

In earlier studies, levels of risk and safety were thought to be important determinants of destination image (Kozak et al., 2007). However, with continuing research on the concept and dimensions of perceived risk, researchers realized that simply using risk to understand the image of recovering destinations had significant limitations (Chew and Jahari, 2014). In short, concepts of a simple linear relationship between risk and image needed to be rethought (Xie et al., 2020). It is true that subsequent empirical studies have confirmed that perceived risk adversely affects the destination image (Xie et al., 2020). Equally, more attention has been paid to the various forms of a crisis. It is suggested that these generate different forms of risk and thus result in different images of a destination. For example, psychosocial and financial risk exerted adverse effects on destination image after the Fukushima Event in Japan, while physical risk directly affected the intention of tourists to travel, but had no significant impact on the destination image (Chew and Jahari, 2014). Hence, we propose the following hypothesis:

H1: Tourists’ risk perception exerts a significant positive impact on negative destination image.

### Tourists’ self-protection behavior

In tourism studies, researchers have applied PMT to examine tourists’ perception of destination risk and protective behavior (Cahyanto et al., 2016; Zheng et al., 2021). Bhati et al. (2021) adapted the PMT framework by proposing health protective behaviors as a mediator of destination health risk images and travel behaviors. Equally, sensitivity to risk shapes tourist willingness to adopt protective behaviors. For example, Majid et al. (2020) analyzed 149 studies from different regions and reported that risk perception was the primary factor determining health and social distancing behaviors, and higher risk perception was positively correlated with compliance with isolation protocols, avoidance of crowds, and support for quarantine measures. Researchers have also found that differences in demographic characteristics (e.g., education, income, and gender) also create variations in tourist protective behavior (Cahyanto et al., 2016; Hotle et al., 2020). Additionally, high levels of perceived risk will mean tourists will actively seek to avoid destinations perceived as dangerous to health (Sönmez and Graefe, 1998; Dolnicar, 2007; Cahyanto et al., 2016). Therefore, we proposed the following hypothesis:

H2: Tourists’ perceived risk exerts a significant positive impact on tourists’ self-protection behavior.

In short, previous studies have established that tourists strongly reject and avoid destinations with negative images (Khan et al., 2017). For example, Kozak et al. (2007) mentioned that infection and terrorist attacks were the two key reasons affecting a destination’s image and changes in travel plans. Based on this, it was hypothesized that:

H3: Negative destination image exerts a significant positive impact on tourists’ self-protection behavior.

### Tourists’ satisfaction

Although the subject of tourism satisfaction is a crucial topic in the field of tourism research (Cohen et al., 2014), and is intimately linked with behavior (Bowen and Clarke, 2002), the definition of tourist satisfaction remains controversial. Scholars study tourist satisfaction from several perspectives. These include expectation- disconfirmation theory (Pizam, 1978; Oliver, 1980), performance models (Kozak, 2000), outcome–input models (Oliver and Swan, 1989), and cognitive-affective concepts (Del Bosque and San Martín, 2008). Numerous empirical studies are based on expectations and performance (Ma et al., 2020). Researchers who support the theory that satisfaction is solely determined by expectations emphasize the role of performance and quality of destination attributes (Bernini and Cagnone, 2014). In turn, expectation theory holds that tourists form certain expectations derived from a long-term collection of information, experience, and destination image cognition. This approach emphasizes the equal significance of the pre-tour and travel process and focuses more on the interaction of individual variables. In a situation where the COVID-19 pandemic has markedly limited and altered the tourism industry, the behavior of tourists largely depends on their perception of safety and risks related to travel activities (Godovykh et al., 2021). Hence, it is apt for this study to measure the formation of tourist satisfaction by including measures of expectations.

However, expectations represent but one variable (Chen et al., 2013). Previous literature includes expectations (Hui et al., 2007), expectation diversity (Kozak, 2000), perceived value (Bradley and Sparks, 2012), emotion (Del Bosque and San Martín, 2008), perceived quality (Kim et al., 2011), the image of a tourism destination (O’Leary and Deegan, 2005), and tourist motivation (Prayag and Ryan, 2012) as determinants of satisfaction. Simultaneously, owing to diverse destination types, different social environments and stages of economic development, the varied physical and mental states of tourists, the environments from whence they came, and so on, enormous differences exist in the contribution of different factors to the entire formation process of satisfaction (Bowen and Clarke, 2002). However, little of this research has been conducted during a tourism crisis. Consequently, this study proposes these measures have a role to play, but need to be contextualized in situations of general risk such as those posed by epidemics and risk of threatening viral transmission.

One theme in past research is the relationship between tourist perception of risk and satisfaction (Jin et al., 2016). In the context of a crisis, risk perception becomes a crucial factor that may determine tourist satisfaction. For example, in a study of international tourists traveling to Beijing, Li et al. (2016) found a significant negative correlation between the risk of experiencing air pollution and satisfaction. In addition, perceptions of risk change with greater travel experience. For example, Xie et al. (2020) demonstrated that the impact of perceived pre-travel risk on satisfaction was significantly mediated by post-travel perceived risks and experience of the destination. Consequently, we proposed the following hypothesis:

H4: Tourists’ risk perception exerts a significant negative impact on tourist satisfaction.

It has been found that the more positive the destination image, the higher the resultant tourist satisfaction (O’Leary and Deegan, 2005; Wang and Hsu, 2010). Similarly, the more negative the assessment of the destination by tourists, the lower the satisfaction (Castro et al., 2007). While these relationships are generally stable for various tourism environments (Bui and Le, 2016; Pramod and Nayak, 2018), in a disaster, negative images may well be the more powerful determinant of satisfaction. Tang (2014) used the 2008 earthquake in Wenchuan, Sichuan, and found that the impact of a negative image on visitor satisfaction was far stronger than any positive image. Accordingly, the following hypothesis is proposed:

H5: Negative destination image exerts a significant negative impact on tourist satisfaction.

For most tourists, if risk exists within a destination, measures can be taken to avoid potential danger, thereby decreasing the potential harm to self (Dolnicar, 2007). Such protective behaviors can generate positive emotional responses and increase overall satisfaction (Lin et al., 2012). As noted by Huang et al. (2020), in the face of risk factors almost impossible to change (e.g., high altitude and cold), encouraging tourists’ self-protection behavior is crucial for improving levels of satisfaction. Based on this, we proposed the following hypothesis:

H6: Tourists’ self-protection behavior exerts a significant positive impact on tourist satisfaction.

### Mediating effects of negative destination image and self-protection behavior

Previous studies reveal that tourist risk perceptions negatively affect destination image (Ruan et al., 2017; Loureiro and Jesus, 2019; Xie et al., 2020), while destination image has also been shown to directly affect satisfaction (Prayag and Ryan, 2012; Su et al., 2020). Given our previous hypothesis regarding the effect of risk perception on satisfaction, we therefore predict that a negative destination image may mediate this relationship. There is evidence that tourists’ risk perceptions trigger their self-protection behaviors under different health crises (e.g., rabies, haze, COVID-19) (Wang et al., 2019; Ruan et al., 2020; Zheng et al., 2021, 2022). The ability of tourists’ preventive behaviors to significantly increase their satisfaction with high-altitude destination tourism has also been confirmed (Huang et al., 2020). Previous studies have focused on the relationship between destination image and tourist behavior (revisit intention, recommendation) (Sirgy and Su, 2000; Lee, 2009; Tavitiyaman and Qu, 2013; Rasoolimanesh et al., 2021), while there has been little research on how destination image affects self-protection behavior. Naturally, we hypothesized that negative destination images may positively influence tourist self-protection behaviors. Therefore, we predict that tourist self- protection behavior may mediate this relationship and that there may be serial multiple mediators involving negative destination images and self-protection behavior. Thus, we proposed the following hypotheses:

H7a: the relationship between tourist perceived risk and satisfaction is mediated through negative destination image.

H7b: the relationship between tourist perceived risk and satisfaction is mediated through self-protection behavior.

H7c: the relationship between tourist perceived risk and satisfaction is mediated through negative destination image and self-protection behavior.

**FIGURE 1:**
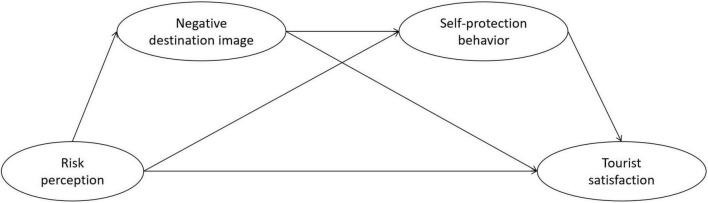
Proposed research model.

Based on the previously discussed hypotheses (H1–H7), we propose the following research model (Figure 1).

## Methodology

Singh et al. (2012) highlighted the academic value of combining quantitative and qualitative methods to study complex tourism phenomena. This study adopted the sequential interpretation approach proposed by Creswell and Clark (2017). First, quantitative data were obtained and analyzed through questionnaire collection, followed by in-depth interview results based on qualitative methods to provide a more comprehensive analysis of the phenomenon under investigation (Creswell and Plano Clark, 2011; McBride et al., 2019).

In the quantitative part of the research, this study used a questionnaire to obtain quantitative research data. The questionnaire consists of two main parts. The first part is a 23-item questionnaire (Appendix A) that measures the perceived risk of COVID-19, destination image, self-protection behavior, and tourist satisfaction. All constructs were assessed using multiple items, with the measurement items derived from interviews and previous studies and slightly modified for this study, using a 7-point Likert scale ranging from 1 = strongly disagree to 7 = strongly agree. The second part of the questionnaire consisted of respondents’ personal information, including gender, age, education, marital status, number of children, occupation, and monthly income level. Our survey followed the procedural recommendations of Podsakoff et al. (2003) regarding respondent anonymity (to minimize assessment concerns and item ambiguity). The questionnaire was first pre-tested online *via* a Tencent online questionnaire to 45 tourism management students and teachers working in the tourism management profession to ensure content validity. The questionnaire was finalized after feedback from the aforementioned people’s revisions and review by academic experts. The field survey was conducted from 1 May to 24 May 2020. The field questionnaire was conducted in Huizhou Ancient Town, Tunxi Old Street, and Chengkan Town in Huangshan City, China; Tianyi Pavilion Scenic Area, Cicheng Scenic Area, and Ningbo Museum in Ningbo; and Giant Panda Breeding and Research Base, Dujiangyan Scenic Area, and Taikoo Li Scenic Area in Chengdu City. The questionnaires were distributed using a convenience sampling method, whereby the research team randomly selected tourists to distribute questionnaires at tourist attractions. Overall, a total of 543 questionnaires were distributed, 486 completed questionnaires were valid.

We conducted in-depth interviews using a snowball sampling technique with 19 tourists (Table 1) filtered based on the following two conditions: (1) respondents must have traveled domestically during the epidemic period (2020 and 2022); and (2) tourists must be at least 18 years old, because of the limitations of epidemic prevention and control. These interviewees were randomly selected based on observations that would meet the needs of this study. We conducted interviews between June and July 2022 and stopped recruiting new interviewee after theoretical saturation was reached. Based on ethical considerations of confidentiality, anonymity, and privacy protection, we confirmed with the interviewees that they all volunteered to be interviewed (Bryman and Bell, 2011). Questions for the interviews were designed based on consideration of variables relevant to the quantitative study (Appendix B). The in-depth interviews enabled an interpretive approach to validate the quantitative findings of the study (Bryman and Bell, 2015), while respondents could easily express their views and add information and statistical analysis that the quantitative data could not convey. We conducted the interviews through Tencent meetings or phone calls. The entire interview was recorded, and the interview process lasted 20–30 min, after which the researcher transcribed it into text. Finally, the research analyzed the content of all 19 interviewees by using content analysis technique.

**TABLE 1:** Information of qualitative study interviewees.

Number	Gender	Profession	Education background
1	Female	Student	Bachelor
2	Female	Student	Secondary studies
3	Female	Employees of private enterprises	Bachelor
4	Male	Employees of state enterprises	Bachelor
5	Female	Civil Servant	Bachelor
6	Female	Student	Bachelor
7	Female	Employees of state enterprises	Bachelor
8	Male	Student	Secondary studies
9	Female	Employees of state enterprises	Bachelor
10	Male	Student	Bachelor
11	Male	Employees of state enterprises	Bachelor
12	Female	Employees of state enterprises	Bachelor
13	Female	Employees of state enterprises	Bachelor
14	Male	Student	Bachelor
15	Male	Employees of private enterprises	Secondary studies
16	Male	Nurse	Bachelor
17	Female	Teacher	Postgraduate
18	Male	Freelancer	Bachelor
19	Male	Freelancer	Bachelor

## Results

### Quantitative study

Of the sample, (shown in Table 2) a small majority of respondents were male (51.9%) and half were aged between 19 and 29 years, while a further 30% were aged between 30 and 49 years. Almost two-thirds possessed a university qualification. About one-quarter worked in the private commercial sector, 17% were students, and 12% of the respondents worked in engineering and professional technical services. Approximately two-thirds self-reported that they received an average income and a quarter self-reported an above-average income. The sample is thought to be reasonably representative of those who tend to holiday reasonably frequently in contemporary China by being younger, well qualified, and of average and above average income.

**TABLE 2:** Demographic characteristics of respondents.

	Category	Frequency	Percentage (%)		Category	Frequency	Percentage (%)
Gender	Male	254	52.3	Occupation	Civil servants	26	5.3
	Female	232	47.7		Staff of enterprises or institutions	109	22.4
Marital status	Unmarried	263	54.1		Private business owner	45	9.3
	Married	211	43.4		Waiter/ salesperson	38	7.8
	Divorce/Separation	12	2.5		Professionals	57	11.7
Age	Under 19	7	1.4		Self-employed	45	9.3
	19–29	244	50.2		Freelancer	44	9.1
	30–39	149	30.7		Student	85	17.5
	40–49	53	10.9		Separation/ Retirement	10	2.1
	50–59	27	5.6		The other	27	5.6
	Above 59	6	1.2	Monthly income level	Far below the average	10	2.1
Educational background	Junior high school and below	27	5.6		Below average	27	5.6
	Senior High School	88	18.1		Average	310	63.8
	University	310	63.8		Above average	128	26.3
	Postgraduate or above	61	12.6		Well above average	11	2.3

Due to the single source of data for this study, potential common method bias (CMB) was possible, so procedural controls were performed, i.e., respondents were invited to fill out the questionnaire in such a way that they were all ensured to be aware of the anonymity. Secondly, statistical control was also considered as the remedy, we tested this by Harman’s single factor test, which means that all items were included in the exploratory factor analysis (EFA). And the result showed that the total variance explained by the four factors was 64.427%, while the variance of first (largest) factor accounted for 24.223% which does not exceed 50%, so we can assume that there is no problem of potential CMB in the data. In addition, the skewness and kurtosis values of all items were examined in this study, and the results showed that the absolute values of skewness were distributed in the range of 0.151–1.227, which is less than 2, and the absolute values of kurtosis was distributed in the range of 0.567–1.532, which is less than 2, both in accordance with the normal assumption.

According to Table 3, the confirmatory factor analysis (CFA) model showed a good fit (χ2/df = 2.101; GFI = 0.925; CFI = 0.964; TFI = 0.958; IFI = 0.965; RMSEA = 0.048; SRMR = 0.056), proving that the measurement model is acceptable. Next, this study examined the reliability of the measurement instrument, which was estimated by two indicators, Cronbach’alpha and composite reliability (CR) value. The distribution of Cronbach’s alpha for all dimensions was 0.797–0.922, which was greater than the ideal value of 0.7, indicating that the scale had good internal consistency, and the lowest CR value among all dimensions was 0.808, which was also higher than the threshold value of 0.7. All factor loadings were greater than 0.6, except for the three items (RP1 = 0.592, NDI6 = 0.545, SPB3 = 0.551). The average variance extracted (AVE) for the negative destination image is slightly below the usual threshold, although a value above 0.36 is acceptable according to Fornell and Larcker (1981), with AVE values above 0.5 for the remaining dimensions. According to the Table 4, the correlation coefficients between all dimensions were less than the square root of the AVE of each dimension, which indicates that the scale has good discriminant validity.

**TABLE 3:** Results of reliability and validity analysis.

Constructs and items		FL	AVE	CR	Cronbach’α
Risk Perception	RP1	0.592	0.526	0.908	0.922
	RP2	0.691			
	RP3	0.686			
	RP4	0.860			
	RP5	0.793			
	RP6	0.749			
	RP7	0.748			
	RP8	0.709			
	RP9	0.667			
Negative Destination Image	NDI1	0.635	0.486	0.848	0.797
	NDI2	0.659			
	NDI3	0.831			
	NDI4	0.725			
	NDI5	0.753			
	NDI6	0.545			
Self-Protection Behavior	SPB1	0.765	0.520	0.808	0.856
	SPB2	0.887			
	SPB3	0.551			
	SPB4	0.636			
Tourist Satisfaction	TS1	0.815	0.651	0.881	0.891
	TS2	0.887			
	TS3	0.785			
	TS4	0.733			

χ^2^/df = 2.101; GFI = 0.925; CFI = 0.964; TFI = 0.958; IFI = 0.965; RMSEA = 0.048; SRMR = 0.056. FL, factor loading; AVE, average variance extracted; CR, composite reliability.

**TABLE 4:** Results of discriminant validity.

Constructs	RP	NDI	SPB	TS	Mean	Std.
RP	**0.725**				5.865	0.866
NDI	0.337**	**0.697**			4.393	0.980
SPB	0.348**	0.315**	**0.721**		5.594	1.050
TS	0.064	−0.233**	–0.035	**0.807**	5.411	0.937

**p < 0.01. The diagonal elements in bold are the squared roots of the AVE. RP, Risk Perception; NDI, Negative Destination Image; SPB, Self-Protection Behavior; TS, Tourist Satisfaction; Mean, Mean value; Std., Standard deviation

We constructed a SEM of the conceptual model through AMOS 24.0 to test the effect of the independent variable (tourists’ risk perception) on the dependent variables (negative destination image, self-protection behavior, and tourists’ satisfaction) using SEM. The results showed (Table 5) that risk perception significantly influenced negative destination image (β = 0.385, *p* < 0.001), thus H1 was supported; risk perception significantly and positively influenced self-protection behavior of tourists (β = 0.196, *p* < 0.001), therefore H2 was supported; risk perception had a significant negative effect on tourist satisfaction (β = –0.213, *p* < 0.001) providing support to H3; negative destination image had a significant positive effect on self-protection behavior (β = 0.290, *p* < 0.001), therefore H4 was supported; negative destination image had a significant negative effect on tourist satisfaction (β = –0.199, *p* < 0.001) which supports H5; self-protection behavior had a significant negative effect on tourist satisfaction had no significant effect (β = 0.110, *p* > 0.05) thus H6 was not supported.

**TABLE 5:** Results of SEM analysis.

Label	Path	Estimate	*SE*	C.R.	*p*	Estimate
H1	RP→NDI	0.395	0.061	6.510	***	0.385
H2	RP→SPB	0.266	0.076	3.521	***	0.196
H3	RP→TS	–0.252	0.063	–3.979	***	–0.231
H4	NDI→SPB	0.383	0.077	4.978	***	0.290
H5	NDI→TS	–0.212	0.064	–3.308	***	–0.199
H6	SPB→TS	0.088	0.045	1.948	0.051	0.110

***p < 0.001.

RP, risk perception; NDI, negative destination image; SPB, self-protection behavior; TS, tourist satisfaction.

Finally, we applied model 6 of the process macro program developed by Hayes to check for indirect effects and used a bias-corrected bootstrap confidence intervals (CIs) method with 5,000 replicate samples (Hanemann and Kanninen, 1996; Arrow, 2001) to assess potential independent and serial mediation effects, and if the indirect effect of 95% CI does not contain 0 then the mediating effect is indicated. Table 6 shows a significant direct effect between risk perception and satisfaction (β = –0.155, 95% CI = –0.231, –0.080) and a significant indirect effect of negative destination image between risk perception and tourist satisfaction (β = –0.054, 95% CI = –0.088, –0.025), with a significant partial independent mediating effect of negative destination image between the two which supports H7a. The indirect effect of risk perception through self-protection behavior on tourist satisfaction was not significant (β = 0.014, 95% CI = –0.001, 0.033), while the path “risk perception → self-protection behavior → tourist satisfaction” did not hold, thus indicating H7b was not supported. The indirect effect of negative destination image and self-protection behavior between risk perception and tourist satisfaction (β = 0.006, 95% CI = –0.000, 0.013), there is no significant chain mediating effect of negative destination image and self-protection behavior between the two, thus H7c was not supported.

**TABLE 6:** Results of mediation analysis.

Path	Effect	BootSE	BootLLCI	BootULCI
Total effect	–0.190	0.036	–0.260	–0.119
Direct effect	–0.155	0.039	–0.231	–0.080
Total indirect effect	–0.034	0.019	–0.071	0.002
RP - > NDI- > TS	–0.054	0.016	–0.088	–0.025
RP - > SPB - > TS	0.014	0.009	–0.001	0.033
RP - > NDI- > SPB - > TS	0.006	0.004	0.000	0.013

RP, risk perception; NDI, negative destination image; SPB, self-protection behavior; TS, tourist satisfaction.

### Qualitative study

Through in-depth interviews, this study attempted to confirm or disconfirm the path hypothesis in the quantitative analysis, and further explain the longitudinal changes in tourists’ psychology and behavior from the outbreak period to the post-epidemic era.

First, in the context of COVID-19, there is a correlation between tourist risk perception and negative destination image. In other words, while assessing travel risks during the epidemic, individuals will also consider the consequences of the epidemic on the local tourism industry. Such consequences are multifaceted. Some tourists believe that the tourism industry in the destination is unable to provide high-quality services during the epidemic, such as the closure of some restaurants and hotels, lower traffic volumes, and limited access to scenic spots. Two quotations further confirm the validity of H1.

*Especially in cultural tourism attractions, such as blocks or ancient towns and villages, because tourists have to keep in contact with residents and tour guides, and its spatial scale is relatively small, like in a block with dense tourists and a large flow of people, we are all worried about being infected. The hotel staff will ask you where you came from, with or without an asterisk in the travel code, whether you had a nucleic acid test done, and whether the nucleic acid report was overdue. (Under the influence of the epidemic) some restaurants may be closed, and dine-in is not allowed, only take-out is allowed, but then the color, presentation, and taste will be affected. The same is true for transportation. Before the epidemic, I thought it would be nice to take a train or a high-speed rail to see the scenery, but now, I may not choose a mode of transportation where so many people share one carriage. (Interviewee 14, male, student)*

*Due to the epidemic, flights or high-speed trains may be reduced, and restaurants may be closed. I went to a small shop in Chengdu a few days ago, I heard that there were a lot of people before, but now there are fewer and fewer people. I’m not sure if it will close down 1 day and I can’t go there to mark, what a pity. I feel that the problems encountered in shopping, accommodation, scenic spots and catering are the same. With fewer tourists, their business and operations are definitely not as good as they used to be. When I go there, if the shopping malls or homestays were closed down, I have less choice. (Interviewee 6, female, student)*

According to PMT, when individuals have a high risk perception, they will actively take measures to mitigate the risk. Especially in the context of an unprecedented pandemic, tourists’ subjective assessment of risk during an epidemic can directly motivate their self-protection behaviors to reduce the probability of infection. Furthermore, many interviewees mentioned that vaccination against COVID-19 can also reduce their risk perception. Two citations further confirm the validity of H2.

*I am still a little worried, and I saw the high-speed train crew are wearing protective masks and protecting very strictly, I will have a sense of wariness, will feel serious and a little dangerous. So I will also wear a mask all the time. (Interviewee 18, male, freelancer)*

*I will take precautions during the trip, including wearing a mask, protective gloves, and so on, which are now essential for travel. When choosing a destination, I will avoid crowded places and choose some natural attractions, like grasslands or mountains. (Interviewee 14, male, student)*

The interview data also supported that risk perception can negatively affect tourist satisfaction. This is because tourists are worried that they may need to be quarantined at their own expense, which not only increases financial risk, but may also affect their travel plans and emotion, and tourist satisfaction is consequently affected. Three quotations further support H3.

*If the policy is imposed uniformly in all cases (in destination), I probably won’t go there (to travel). If there is an epidemic, I will worry about the impact on the mood of happiness. The satisfaction depends on whether the local policy is reasonable. The epidemic is not under the control of tourists. (Interviewee 5, female, civil servant)*

*Originally, I planned to go to Hangzhou, but the local epidemic prevention policy required 7 days at home and three nucleic acid tests. So we had to change our trip to Nanjing. This had a great impact on my travel plans, because I have been to Nanjing last year and we had no plans to Nanjing. I felt that my satisfaction with the whole trip suffered as it did not live up to my previous expectations for this trip. (Interviewee 19, male, freelancer)*

*At any time, I have to check whether there are new confirmed COVID-19 cases on my mobile phone, and I feel unhappy and scared. For example, if I am in Jinan, I am afraid that there will be a new confirmed case in Jinan suddenly, and then I cannot go back to Hengshui. I am worried that the local epidemic will affect my return trip. (Interviewee 10, male, student).*

The negative destination image leads tourists to increase their self-protection behavior. Specifically, when tourists perceive a more negative image of the destination, it implies that the destination is more severely affected by the epidemic. This likewise motivates tourists to adopt effective coping strategies to avoid being personally affected by the epidemic, and these strategies include leaving the tourist destination immediately. A quotation further confirms the validity of H4.

*Because I went to Shanghai just before the outbreak of the epidemic in Shanghai, my original plan at that time was to stay in Shanghai for one more day, but when I saw the situation of the epidemic in Shanghai, we left right away. Especially there was an isolation site below the hotel where I was staying, and the whole hot pot restaurant was closed due to someone being tested positive there. As I saw this situation, I was worried about being infected and left Shanghai the next morning. (Interviewee 19, male, freelancer)*

When destination image is damaged due to the epidemic, tourist attractions are unable to provide the same quality of visiting and experiencing for tourists, as usual, resulting in a poor travel experience and quality, which in turn affects overall satisfaction. It is worth mentioning that tourists perceive the local epidemic prevention policy as one of the factors influencing negative destination image. Specifically, when an epidemic breaks out in a destination, the government will require scenic spots and other high-traffic areas immediately cease receiving tourists. Although uniform policies are an effective way to stop the rapid spread of the epidemic, for tourists, this disrupts their travel plans and schedules to a large extent. Moreover, if a local government or tourism-related departments do not provide timely solutions or compensation for tourists, this will eventually lead to decreased tourist satisfaction. Two quotations further confirm the validity of H5.

*During the national holiday, a group of tourists just arrived in Xinjiang when there was an epidemic, so there emerged many unpleasant things. Because they had already made many travel tips before going, Xinjiang is a low-risk area, there is no policy restriction and they can go directly to travel. But the epidemic suddenly appeared that day, the scenic spot was directly closed on the spot, a large number of people gathered at the entrance of the Narathi grassland, everyone had to stay on the same day, and some hotels could not afford to receive them. In that situation at that time, there will be complaints and dissatisfaction, which will produce various psychological emotions. (Interviewee 15, male, employee)*

*I must be very uncomfortable, because first is the loss of money, and then (mentally also) I am very tired and depressed, which will affect my (psychological) state. (Interviewee 4, male, employee)*

It is worth mentioning that the correlation between self-protection behavior and satisfaction was confirmed. This conclusion is contrary to the results obtained from the quantitative data, which probably occurred because, from a longitudinal perspective, tourists will consciously take personal protective measures during travel, such as wearing masks. In fact, these self-protection behaviors are, to a certain extent, initiated by the authorities and society for the public to adopt, and gradually this initiative has become a habit, and tourists have adapted to take personal precautions. Thus, when tourists take self-protection measures, it helps alleviate their psychological concerns and further enhance their satisfaction with the tourism experience. Two quotations further confirm the validity of H6.

*I will prepare some masks, carry disinfecting wipes, and wipe my hands before eating. (These measures are) indeed more inconvenient, but now the inconvenience has become a habit. It is a good idea to show the QR code everywhere you go, we definitely are uncomfortable at first and find it troublesome, but when it becomes a part of our life, we will feel that it is a good thing for everyone. We all show the health code and there is no yellow or red code, it makes me feel at ease and everyone else also feels at ease, I think it is okay. (Interviewee 1, female, student)*

*I think if I decide to travel then, I will definitely put myself in good protection. This will make my travel experience better, and protect not only myself but also others in the attraction. (Interviewee 16, male, nurse)*

## Conclusion

Until now, COVID-19 has not been effectively controlled globally, and its impact on the tourism industry of various countries continues. Increasingly, scholars are paying attention to COVID-19 research on the tourism industry and tourist behavior (Kaushal and Srivastava, 2021; Zheng et al., 2021). The study of the impact of tourists’ risk perception of COVID-19 on satisfaction is helpful for tourism enterprises to formulate scientific marketing strategies and for the recovery of the destination tourism economy. We conclude that tourists’ risk perception, negative destination image, and self-protection behavior are factors that significantly affect their satisfaction during the COVID-19 pandemic. Due to the travel risks and concerns associated with COVID-19, tourists have a negative view of the terrain image of the destination, and they feel an urgent need to perform self-protection behavior during travel. Interestingly, tourists’ self-protection behavior does not affect their satisfaction, and the correlation between the two has been confirmed in the qualitative analysis. In addition, an important finding of our research is that risk perception can also reduce tourist satisfaction through the mediating variable of negative impact on tourist destination image.

### Theoretical implications

The theoretical contributions of this paper are mainly reflected in the following aspects: first, unlike previous discussions of tourist satisfaction that focused on a single factor or multiple factors (Yüksel and Yüksel, 2007; Prebensen and Xie, 2017; Xie et al., 2020), this study evaluates the effect of risk perception of COVID-19 on tourist satisfaction in the 21st century, which is the largest and longest-lasting public health event in human history. At present, research on psychological impact and behavior in the context of COVID-19 mainly focuses on how people feel and cope with risks and their effect on behavior (Yang et al., 2021). This research not only enriches the research content of tourist satisfaction but also expands the application scope of tourist risk perception research. Although scholars have conducted a fruitful quantitative evaluation of the relationship between risk perception and satisfaction (Tavitiyaman and Qu, 2013; Sohn et al., 2016) under the background of the COVID-19, the antecedent variable of tourist satisfaction is still unknown. Specially, risk perception had a significant negative effect on tourist satisfaction, that is, the more risk that tourists perceive, the lower their satisfaction, validating existing research findings (Tavitiyaman and Qu, 2013; Olya and Al-ansi, 2018). For example, tourist satisfaction is significantly reduced when tourists experience mental fatigue, physical discomfort, or emotional instability such as anxiety or nervousness during tourism, or when the quality of the travel experience decreases due to epidemics, and so on (Gallarza and Saura, 2006). Negative destination image had a significant negative effect on tourist satisfaction, which is consistent with several previous studies (Chi and Qu, 2008; Prayag, 2009; Wang and Hsu, 2010; Tavitiyaman and Qu, 2013).

Second, this study also explored the mechanisms mediating the role of negative destination images and self-protection behaviors between risk perceptions and satisfaction. Specifically. Risk perception significantly influenced negative destination image, supporting previous studies (Kozak et al., 2007; Mlozi, 2014; Kani et al., 2017; Nazir et al., 2021). Therefore, the greater a tourist’s risk perception, the more pronounced negative destination image, for example, when a tourist fears that an epidemic will affect the quality of tourism, this will reduce the enjoyment of the trip, or cause a change in travel plans during the trip. Risk perception significantly influenced tourist self-protection behavior, reinforcing previous findings (Kozak et al., 2007; Zheng et al., 2021). If they fear contracting an infection or being quarantined upon return, tourists will take more effective epidemic protection measures to adequately safeguard themselves (Zheng et al., 2021). Negative destination image had a significantly positive effect on self-protection behavior, validating previous findings (Van Herck et al., 2004; Bratić et al., 2021). Due to the restricted flow in destination areas and the decrease in tourist traffic, some restaurants and tourism goods stores may also close, at least temporarily, which would significantly reduce tourist satisfaction. For epidemic prevention and control purposes, tourists are also required to strictly follow the epidemic prevention rules and adopt protective measures such as wearing masks and keeping a distance of one meter between tourists (Humagain and Singleton, 2021). In this context, the mediating effect of destination image complements the results of two existing studies on the relationship between risk perception and tourist satisfaction (Yüksel and Yüksel, 2007; Li et al., 2016; Sohn et al., 2016; Swart et al., 2018), that is, the perception of risk in a tourist destination can accelerate the reduction of tourist satisfaction through a negative destination image, validating the results of existing studies (Nouri et al., 2018). According to our findings, the mediating effect of negative destination image validates the existing hypothesis, since tourism, as an industry with significant mobility characteristics (Hannam et al., 2014), was hit hard by the pandemic in its destination image. The inadequate supply of catering and transportation in the destination, the closure of some scenic spots, the closing of tourist stores, and the decline in the quality of hotel services have made the travel experience of tourists less enjoyable. Therefore, the negative perception of destination image further reduces tourist satisfaction. Furthermore, this paper introduces the theory of protection motivation to explore the impact of tourists’ self-protection behavior on their satisfaction, while at the same time, the negative image of the destination and confirmed that both are essential determinants of tourist satisfaction, validating existing studies (Alrawadieh et al., 2019; Lu and Wei, 2019).

Third, inspired by the mixed-methods research on tourism and the hotel industry conducted by Truong et al. (2020), we used a sequential explanatory mixed-method approach and combined it with PMT in this study. The quantitative results of this paper further clarify the pathway of risk perception on tourist satisfaction, and the interview results we collected strengthen the validity of the SEM constructed in this paper. Our findings also showed that self-protection behavior had no significant effect on tourist satisfaction. This indicates that engaging in self-protection behavior was unrelated to tourist satisfaction. This finding is not consistent with previous studies (Li et al., 2016; Huang et al., 2020), which showed a significant positive relationship between these two variables. However, it validates the finding of Huang et al. (2020). In conclusion, self-protection behavior during tourism is only necessary to avoid self-infection and prevent the spread of COVID-19, which is a necessary measure taken by tourists traveling away from home, a new normal for epidemic prevention and control in the post-epidemic era, and necessary to implement the destination government’s epidemic prevention and control policy. Therefore, some issues that affect tourist satisfaction beyond the variables in the quantitative research (e.g., epidemic prevention and control policies in various regions, COVID-19 vaccination) have also been recognized.

### Managerial implications

The results of this study show that tourist risk perception and negative image of the destination are factors influencing tourist satisfaction. To further reduce the degree of tourists’ risk perception, local governments as tourist destinations, based on the premise of strengthening epidemic prevention and control, should introduce flexible and humane policies (e.g., the 50% restriction on personnel in indoor closed public places will be removed, and the residence and travel history in medium and high-risk areas will be adjusted from 14 to 7 days), increase financial subsidies, and actively issue tourism consumption vouchers. Tourism-related departments encouraging tourists to accept and adapt to COVID-19 is crucial, such as actively providing free psychological counseling services for visitors.

Chi and Qu (2008); Albaity and Melhem (2017), and Prayag et al. (2017) and other scholars discussed how to improve the target terrain image further to improve satisfaction. Therefore, in the uncertain future of COVID-19, the destination management department should further enhance the image of the tourist destination and try its best to eliminate the negative impact of the epidemic. Therefore, all industries should develop in a coordinated manner. For example, the cancelation of the “asterisk” marking policy of communication travel cards has accelerated the recovery of the market. All departments need to actively adapt to market demand and fully integrate tourism factor resources. At the same time, we should do an excellent job in skill training for tourism-related practitioners, pay close attention to the dynamics of the epidemic, adjust tourism products in time, make plans, and improve emergency response capabilities.

Tourist satisfaction is an important concept of tourism marketing (Chew and Jahari, 2014; Michalkó et al., 2015; Sohn and Yoon, 2016), and tourism marketing departments should still consider improving tourist satisfaction as a core task during the epidemic. Precisely, depending on the epidemic risk situation, scenic spots should adjust in a timely manner the degree of passenger flow restriction and provide tourists with real-time information about the scenic area (high and low peak time periods in the scenic spot, scenic spot heat maps, etc.), epidemic prevention and reassurance table in the picturesque place (disinfection and sterilization records of scenic spot facilities and health conditions of service personnel), and set up intelligent equipment to check tourists’ health codes and travel cards, so as to shorten the waiting time of tourists in line. In addition, managers should also improve the transportation convenience of tourist attractions, reduce the travel costs of tourists, and try to provide contactless services to tourists.

### Future research and limitations

Finally, we should acknowledge some limitations of our study. Considering the findings related to risk perception and tourist satisfaction, the findings of this study cannot be generalized and applied to all tourist destinations, because the risk level of the epidemic differs in each tourist destination, and the related epidemic prevention measures and their effectiveness varies. Furthermore, this study only explored the factors influencing tourist satisfaction at the micro level, although we learned from the qualitative interviews that the epidemic prevention policies formulated by the government also influenced tourist satisfaction to a certain extent. Therefore, future studies can expand the in-depth interview sample to increase the factors influencing satisfaction at the macro level (government and society) to further enrich the qualitative findings and better verify the quantitative conclusions. Third, future studies can consider incorporating theories such as cognitive assessment theory and consider more variables such as antecedent variables such as tourist trust, fear of the epidemic, and resilience, as well as outcome variables such as tourist loyalty into the model to better explain tourist behavior under the context of the COVID-19 epidemic.

## Data availability statement

The raw data supporting the conclusions of this article will be made available by the authors, without undue reservation.

## Author contributions

BZ and L-EW: methodology, software and validation, formal analysis, and original draft preparation. BZ, SL, and L-TW: writing—review and editing. Y-XW and SL: visualization. BZ: supervision. L-EW: funding acquisition. All authors read and agreed to the published version of the manuscript.

## Appendix A

**Table:**

**Risk perception** (Peng and Xiao, 2018; Khan et al., 2020; Shin and Kang, 2020; Zhan et al., 2022)	
1	I am worried that my companions and I will be infected
2	I am worried about being quarantined when I return to my home
3	The epidemic made me feel tired easily
4	The epidemic made me feel sick (dizziness, panic, etc.)
5	The epidemic made me feel emotionally unstable (tension, anxiety, etc.)
6	I am worried that the epidemic will affect the quality of travel
7	I am worried that the epidemic will reduce the fun of travel
8	I am worried the epidemic may change my travel plans
9	I am worried that the tourist destination cannot provide value-for-money services
**Negative destination image** (Peng and Xiao, 2018; Neuburger and Egger, 2021)	
1	The epidemic made the catering business of the selected destination unable to operate normally
2	The epidemic made some of the scenic spots in the selected destination close
3	The epidemic made transportation in the selected destination inconvenient
4	The epidemic made hotel accommodation in the selected destination poor quality
5	The epidemic has prevented some stores in the selected destination from opening
6	The epidemic has affected the tourist image of the selected destination
**Self-protection behavior** (Wong and Yeh, 2009; Burns et al., 2017; Peng and Xiao, 2018; Sánchez-Cañizares et al., 2021)	
1	I will take strict protective measures when traveling during the epidemic
2	I prefer to travel to destinations where the epidemic is under control
3	Due to the epidemic situation, I may shorten the length of stay at the destination
4	I will not travel to a tourist destination during the epidemic
**Tourist satisfaction** (Lee et al., 2011; Li et al., 2016; Wang et al., 2017)	
1	Overall, I am satisfied with this trip to the selected destination
2	It was in line with my expectations before the trip
3	It was similar to my ideal tourist destination
4	It was a good choice to travel to the selected destination
	

### Appendix B

**Interview outline**

(1) What do you think are the effects of COVID-19 on people’s health?

(2) What concerns do you have when traveling during an epidemic?

(3) Do these concerns affect your level of satisfaction with the places you travel to? Please tell us why.

(4) What negative impressions do you think the epidemic will have on the transport, dining, shopping, accommodation, and scenic spots of the tourist destination?

(5) Do these negative impressions have an impact on your satisfaction with the destination? Please also explain why.

(6) What protective measures do you take when traveling during the epidemic? Did the epidemic in your destination make you think of ending your trip early or reducing the length of your stay? Or would you prefer to travel to a safer destination?

(7) Do these thoughts or precautions affect your satisfaction with the destination?

(8) Would the image of the destination prompt you to take precautions? Or leave the destination early? Or to travel to a safer place?
